# Development and Internal Validation of Fatty Liver Prediction Models in Obese Children and Adolescents

**DOI:** 10.3390/jcm10071470

**Published:** 2021-04-02

**Authors:** Giorgio Bedogni, Sofia Tamini, Diana Caroli, Sabrina Cicolini, Marco Domenicali, Alessandro Sartorio

**Affiliations:** 1 Clinical Epidemiology Unit, Liver Research Center, 34012 Basovizza, Italy; 2 Istituto Auxologico Italiano, IRCCS, Experimental Laboratory for Auxo-Endocrinological Research, 28824 Verbania, Italy; s.tamini@auxologico.it (S.T.); s.cicolini@auxologico.it (S.C.); d.caroli@auxologico.it (D.C.); sartorio@auxologico.it (A.S.); 3 Department of Medical and Surgical Sciences, Alma Mater Studiorum University of Bologna, 40138 Bologna, Italy; marco.domenicali@gmail.com; 4 Internal Medicine, S. Maria delle Croci Hospital, 48121 Ravenna, Italy; 5 Istituto Auxologico Italiano, IRCCS, Division of Auxology and Metabolic Diseases, 28824 Verbania, Italy

**Keywords:** cross-sectional study, obesity, children, adolescents, diagnostic techniques and procedures, fatty liver

## Abstract

To develop predictive models of fatty liver (FL), we performed a cross-sectional retrospective study of 1672 obese children with a median (interquartile range) age of 15 (13–16) years. The outcome variable was FL diagnosed by ultrasonography. The potential predictors were: (1) binary: sex; (2) continuous: age, body mass index (BMI), waist circumference (WC), alanine transaminase (ALT), aspartate transaminase, gamma-glutamyltransferase, glucose, insulin, homeostasis model assessment of insulin resistance (HOMA-IR), HDL-cholesterol, LDL-cholesterol, triglycerides, mean arterial pressure, uric acid, and c-reactive protein; (3) ordinal: Pubertal status. Bootstrapped multivariable logistic regression with fractional polynomials was used to develop the models. Two models were developed and internally validated, one using BMI and the other using WC as the anthropometric predictor. Both models included ALT, HOMA-IR, triglycerides, and uric acid as predictors, had similar discrimination (c-statistic = 0.81), and were similarly well calibrated as determined by calibration plots. These models should undergo external validation before being employed in clinical or research practice.

## 1. Introduction

The prevalence of fatty liver (FL) is increasing worldwide mostly because of the current epidemics of obesity [1,2]. A recent metanalysis performed in subjects aged 1 to 19 years reported a prevalence of FL of 2.3% in normal-weight, 12.5% in overweight, and 36.1% in obese children [2]. Even if the natural history of FL in children is largely unknown and cause–effect relationships between FL and associated diseases are hard to prove with the present evidence base [3], it is reasonable to assume that FL persisting from childhood to adulthood may have harmful hepatic and extrahepatic consequences [4,5,6,7].

FL is operationally defined as visible steatosis in more than 5% of hepatocytes at liver biopsy or as an intrahepatic triglyceride content of at least 5.6% at magnetic resonance spectroscopy (MRS) or magnetic resonance imaging (MRI) [8]. However, the most common method used to diagnose FL in clinical practice and epidemiological research is liver ultrasonography (LUS) [8,9]. When LUS is not available, the presently suggested method to diagnose FL in adults is the calculation of surrogate indices of FL [8].

The use of surrogate indices of FL is becoming increasingly popular in adults [10]. However, very few data are available to date on surrogate indices of FL in children and adolescents. In a cross-sectional study, performed at our Center in 2007 on 267 subjects aged 8 to 18 years and with a body mass index (BMI) > 90th percentile for age, we found that BMI, alanine transaminase (ALT), uric acid, insulin during oral glucose tolerance testing (OGTT), and glucose during OGTT, contributed independently to FL [11]. Although these variables were potential contributors to a multivariable surrogate index of FL, we did not develop a prediction model, mostly because of the low number of available children [11]. Surprisingly, only one study was performed to date with the aim of developing a prediction model for FL in children and adolescents. In 2011, Hosseini et al. developed a prediction model of FL based on sex, age, BMI, waist circumference (WC) and triglycerides in a sample of 962 Iranian subjects aged 6 to 18 years [12]. Unfortunately, Hosseini et al. [12] had not available measurements of liver enzymes, uric acid, insulin, and c-reactive protein (CRP), which are potential predictors of FL [11,13,14].

Insulin was the strongest multivariable predictor of FL in the general adult population of the Dionysos Nutrition and Liver Study [15,16]. However, when we used the data from the Dionysos Nutrition and Liver Study to develop the fatty liver index (FLI), we did not take insulin into account because its measurement was not routinely performed [16]. Nowadays, insulin is almost routinely measured in obese patients and in those with FL, and it will be increasingly so if the newly proposed definition of metabolic (dysfunction) associated fatty liver disease (MAFLD) will replace the more traditional separation of FL into non-alcoholic fatty liver disease (NAFLD) and alcoholic fatty liver disease (AFLD) [13]. To diagnose MAFLD, one has in fact to calculate the homeostasis model assessment of insulin resistance (HOMA-IR), which is obtained from fasting insulin and glucose [17]. The diagnosis of MAFLD requires also the measurement of CRP [13], which may therefore become more common in patients with FL.

The aim of the present cross-sectional study was, therefore, to develop and internally validate multivariable prediction models of FL for obese children taking into account anthropometry, the components of the metabolic syndrome, liver enzymes, insulin and glucose alone and combined into HOMA-IR, uric acid, and CRP. The study was reported following the “Transparent reporting of a multivariable prediction model for individual prognosis or diagnosis” (TRIPOD) guidelines (Appendix A).

## 2. Subjects and Methods

### 2.1. Source of Data

The development and internal validation of the multivariable models for the prediction of FL were performed using an already available dataset of obese children followed at our tertiary care center for the treatment of pediatric obesity [18]. The study was approved by the Ethical Committee of the Istituto Auxologico Italiano (research project code 1C021_2020, acronym BILOB) and was conducted in accordance with the Declaration of Helsinki. Written informed consent to participate in the study was obtained from the subjects aged 18 years or from the legal representatives of those aged < 18 years.

### 2.2. Participants

All the children were admitted to the Clinic to undergo a short-term structured multidisciplinary weight-loss program and underwent the assessment of the outcome and the predictors before starting such program. The inclusion criteria were: (1) Caucasian ethnic group, (2) age ≤ 18 years, (3) BMI ≥ 95th percentile for age according to Italian growth charts [19], and (4) availability of LUS. The exclusion criteria were: (1) Genetic or syndromic obesity, (2) treatment with any drug, (3) alcohol intake (any quantity), and (4) hepatitis B virus (HBV) or hepatitis C virus (HCV) infection. All children underwent a detailed clinical history and physical examination. Alcohol intake was assessed by interview. Pubertal status was classified by a pediatric endocrinologist in 5 stages according to Tanner [20]. Weight and height were measured following international guidelines [21]. WC was measured at the midpoint between the last rib and the iliac crest. BMI was calculated as weight (kg)/height (m)^2^. Standard deviation scores (SDS) of weight, height and BMI were calculated using Italian growth charts [19]. Performed blood tests included: (1) Alanine transaminase (ALT), (2) aspartate transaminase (AST), (3) gamma-glutamyltransferase (GGT), (4) glucose, (5) insulin, (6) cholesterol, (7) HDL-cholesterol, (8) LDL-cholesterol, (9) triglycerides, and (10) CRP. All blood tests were performed in the fasting state and evaluated using a Cobas 6000 analyzer (Roche Diagnostics, Monza, Italy). Systolic (SBP) and diastolic blood pressure (DBP) was measured using a sphygmomanometer following international guidelines. Mean arterial pressure (MAP) was calculated as SBP*(1/3) + DBP*(2/3). The metabolic syndrome was diagnosed using the criteria of the International Diabetes Federation [22]. HOMA-IR was calculated as (insulin*glucose)/405 [17].

### 2.3. Outcome

The outcome was FL diagnosed by LUS [11,23]: (1) Normal liver was defined as the absence of liver steatosis or other liver abnormalities; (2) mild FL as the presence of slight bright liver or hepatorenal echo contrast without intrahepatic vessels blurring and no deep attenuation; (3) moderate FL as the presence of mild bright liver or hepatorenal echo contrast without intrahepatic vessel blurring and with deep attenuation; (4) severe FL as diffusely severe bright liver or hepatorenal echo contrast, with intrahepatic vessels blurring (no visible borders) and deep attenuation without visibility of the diaphragm. For the present analysis, FL was coded as 0 = normal liver and 1 = any degree of FL. Because of the exclusion of any degree of alcohol intake and of HBV and HCV infections (see Section 2.2), FL as determined by the present study is synonym with NAFLD. One should note, however, that our threshold for alcohol intake (0 g/day) is much lower than that currently used to define NAFLD (<30 g/day in men and <20 g/day in women) [8].

### 2.4. Predictors

The following potential predictors were considered: (1) binary: Sex (0 = female; 1 = male); (2) continuous: age (years), BMI (kg/m^2^), WC (cm), ALT (U/L), AST (U/L), GGT (U/L), glucose (mg/dL), insulin (μU/mL), HOMA-IR (dimensionless), HDL-cholesterol (mg/dL), LDL-cholesterol (mg/dL), triglycerides (mg/dL), MAP (mm Hg), uric acid (mg/dL), and CRP (mg/l); (3) ordinal: Pubertal status (5 Tanner stages). The predictors included all the continuous components of the metabolic syndrome, i.e., WC, glucose, HDL-cholesterol, triglycerides, and blood pressure [22]. MAP was used as measurement of blood pressure to avoid problems of multicollinearity stemming from having SBP and DBP in the same model [24]. The predictors included liver enzymes (ALT, AST, GGT), which have been associated with FL in children [11]. As discussed in detail in the Introduction, insulin was included among the predictors because it was the most effective multivariable predictor of FL in the Dionysos Nutrition and Liver study [16], and because its measurement is required to calculate HOMA-IR, which is needed to diagnose MAFLD [13]; CRP was included because it is similarly required to diagnose MAFLD [13]; lastly, uric acid was included because it has been reported as a promising predictor of FL [14].

### 2.5. Sample Size

This is a cross-sectional retrospective study performed on an already available dataset of 1672 children and adolescents (see Section 2.1). The present sample size calculation was therefore post-hoc. For the reasons discussed at Section 2.4, we were interested to evaluate the *multivariable* association of fatty liver with the following predictors: (1) sex, (2) age, (3) pubertal status, (4) BMI, (5) WC, (6) ALT, (7) AST, (8) GGT, (9) glucose, (10) insulin, (11) HOMA-IR, (12) HDL-cholesterol, (13) LDL-cholesterol, (14) triglycerides, (15) MAP, (16) uric acid, and (17) CRP. This amounts to considering 20 effective predictors, 4 of which are needed to code the ordinal 5-level pubertal status. Using the criteria of Riley et al. [25,26], we calculated that 1535 children were needed to estimate a Cox-Snell R^2^ of 0.11 with a prevalence of 38% of the outcome, under the expectation of (1) a shrinkage of predictor effects < 10%, (2) a difference of 5% in the model apparent and adjusted Nagelkerke R^2^ value and, (3) an estimation within 5% of the average outcome risk in the population. Following the suggestion of Riley et al. [25,26], we choose the lowest vale of Cox-Snell R^2^ that we detected in our experience at developing multivariable models for the prediction of fatty liver. We also ignored likely multicollinear terms, e.g., age and pubertal status [18], that we expected to remove in the model development phase [24,27], to err on the side of including more subjects for the prediction, as we already had a large dataset at our disposal. The available sample size of 1672 children was thus more than enough to develop the models of interest.

### 2.6. Missing Data

There were no missing data.

### 2.7. Statistical Analysis

Most continuous variables were not Gaussian-distributed, and all are reported as median (50th percentile) and interquartile range (25th and 75th percentiles). Discrete variables are reported as the number and proportion of subjects with the characteristic of interest. As suggested by Royston and Sauerbrei [27,28], we examined the Spearman correlation matrix between the outcome and the potential predictors before embarking into multivariable modeling and kept only one of the variables of highly correlated clusters, as defined by a Spearman’s rho > |0.60|, they key criterion being its clinical availability. The importance and the functional form of the predictors was evaluated using degree 2 fractional polynomials coupled to logistic regression in 1000 bootstrap samples with replacement [27,28,29]. Only predictors with a bootstrap inclusion factor (BIF) ≥ 66%, i.e., occurring in more than two thirds of bootstrap samples, were included in the final models. The 95% confidence intervals (95%CI) of the predictors of the final models were calculated using bootstrap in 1000 samples with replacement. Discrimination was evaluated using Harrell’s c-statistic, which is equivalent to the area under the receiver operating characteristic curve [30]. As measures of model fit, besides Cox-Snell R^2^ and Nagelkerke R^2^, we calculated the Akaike information criterion (AIC) and the Bayesian information criterion (BIC) [23]. Internal calibration was evaluated using Van Calster’s 3-level hierarchy [31,32]: (1) “Mean calibration”, which compares the observed event rate with the average predicted risk; (2) “weak calibration”, which tests whether the calibration slope is 1 and the calibration intercept is 0; (3) “moderate calibration”, which uses a calibration plot with a superimposed locally weighted scatterplot smoother to test whether the predicted risks correspond to the observed event rates [33]. The 95%CI of the calibration slope and intercept were calculated using bootstrap on 2000 samples with replacement [31]. Statistical analysis was performed using Stata 16.1 (Stata Corporation, College Station, TX, USA) with the pmsampsize module [34], and R 4.0.4 (R Core Team 2021, Vienna, Austria) with the val.prob.ci.2 function [31]. R code was run from within Stata using the rcall package [35].

## 3. Results

### 3.1. Study Population

Table 1 gives the measurements of the 1672 study subjects. They had a median (IQR) age of 15 (13–16) years, ranging from 5 to 18 years, and most of them (748, 45%) were postpubertal. FL was detected in 642/1672 (38%, 95%CI 36 to 41%) of the study subjects.

### 3.2. Selection of Predictors for Multivariable Modeling

As suggested by Royston and Sauerbrei [27,28], we examined the Spearman correlation matrix between the outcome and the potential predictors before embarking into multivariable modeling (Table 2).

Only one of the variables pertaining to highly correlated clusters was kept, as detected by a Spearman’s rho > |0.60|. Age and pubertal status were highly correlated (rho = 0.82). Age was kept for multivariable modeling because the assessment of pubertal status requires a pediatric endocrinologist, rendering a prediction model including pubertal status not usable in standard clinical practice. BMI and WC were highly correlated (rho = 0.77). Because we aimed at detecting whether the single components of the metabolic syndrome contribute to FL and WC is a component of the metabolic syndrome, we decided to develop two distinct multivariable models, one using BMI and the other using WC as the anthropometric predictor. It should be noted that, in the present study, we used BMI (kg/m^2^) as predictor instead of BMI (SDS), which was used in our previous study [11]. This was done because “raw” BMI is not dependent on any given reference chart and allows a more direct comparison with WC, which was not evaluated in our previous study [11]. ALT was highly correlated with AST (rho = 0.81) and GGT (rho = 0.61). ALT was kept for multivariable modeling because it is more hepatospecific than AST, and because GGT is as a second-level exam in most centers. Lastly, HOMA-IR and insulin were correlated so strongly (rho = 0.98) to be considered synonyms for modeling purposes. We kept HOMA-IR for multivariable modeling instead of insulin because of its pathophysiological significance and because it is presently required for the diagnosis of MAFLD [13]. Glucose was not entered into the multivariable models because it is already included into HOMA-IR (See Section 2.2).

### 3.3. Multivariable Modeling Strategy

Based on the above findings, we decided to evaluate two distinct multivariable models, one using BMI and the other using WC as the anthropometric predictor. Both models included sex, age, ALT, HOMA-IR, HDL-cholesterol, LDL-cholesterol, triglycerides, MAP, uric acid, and CRP as potential predictors. The importance and the functional form of each predictor was evaluated using degree 2 fractional polynomials in 1000 bootstrap samples with replacement [27,28,29]. The bootstrap inclusion fraction (BIF) and the functional form of the fractional terms 1 (BIF-1) and 2 (BIF-2) of the polynomials of the predictors are given in Table 3. Only predictors with BIF-1 ≥ 66%, i.e., selected in at least two thirds of bootstrap samples, were considered for inclusion in the final multivariable models.

As the BMI-based multivariable model is concerned, ALT had the highest BIF-1 (100%), followed by BMI (98%), age (95%), HOMA-IR (95%), uric acid (79%) and triglycerides (74%). All variables were selected as linear except for ALT and HOMA-IR. A second fractional polynomial term was needed for ALT (BIF-2 = 90%), including ALT^−2^ and ALT^−1^, and for HOMA-IR (BIF-2 = 51%), including HOMA-IR and HOMA-IR^2^. 

As the WC-based multivariable model is concerned, ALT had the highest BIF-1 (100%), followed by HOMA-IR (99%), age (91%), WC (90%), uric acid (87%), and triglycerides (70%). All variables were selected as linear except for ALT and HOMA-IR. A second fractional polynomial term was needed for ALT (BIF-2 = 89%), including ALT^−2^ and ALT^−1^, and for HOMA-IR (BIF-2 = 52%), including HOMA-IR and HOMA-IR^2^.

### 3.4. Multivariable Models

The final multivariable models are given in Table 4. The corresponding regression equations are given in Appendix B.

**Figure 1:**
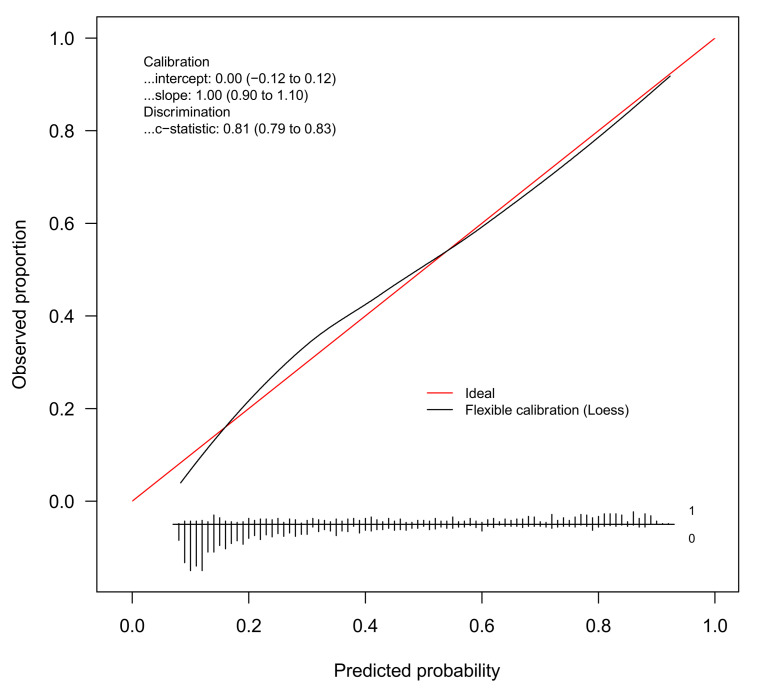
Internal calibration plot for the diagnosis of fatty liver from the BMI model. Abbreviation: loess = locally estimated scatterplot smoother.

The discrimination of the BMI model was good (c-statistic = 0.81, 95%CI 0.79 to 0.83). Figure 1 gives the calibration plot of the same model. The average expected rate of fatty liver (38%, 95%CI 36% to 40%) equaled the average observed rate (38%, 95%CI 36% to 41%), showing a satisfactory mean calibration. At logistic calibration, the average calibration slope was 1 and the average intercept was 0, showing a satisfactory weak calibration. Lastly, the examination of calibration plots showed an acceptable profile of moderate calibration (Figure 1).

**Figure 2:**
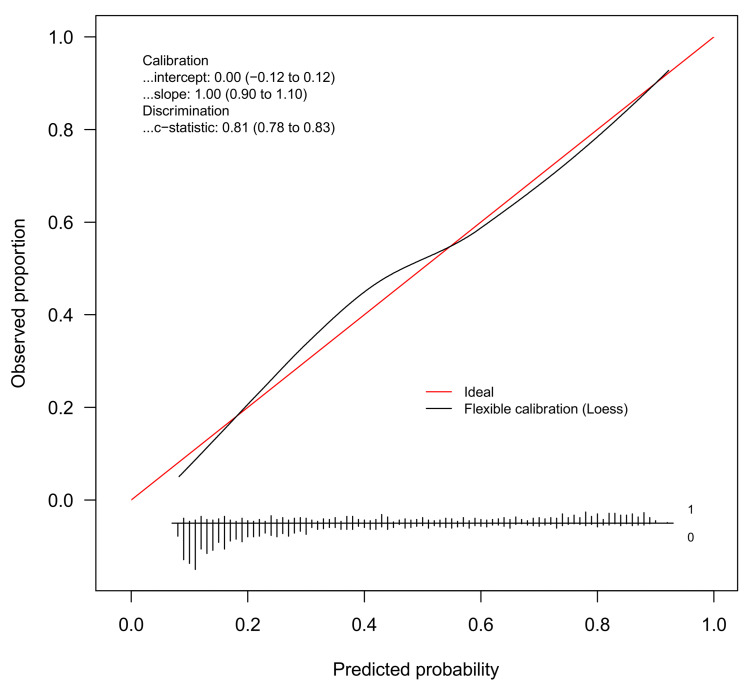
Internal calibration plot for the diagnosis of fatty liver from the WC model. Abbreviation: loess = locally estimated scatterplot smoother.

The discrimination of the WC Model was good (c-statistic = 0.81, 95%CI 0.78 to 0.83). Figure 2 gives the calibration plot of the same model. The average expected rate of fatty liver (38%, 95%CI 36% to 40%) equaled the average observed rate (38%, 95%CI 36% to 41%), showing a satisfactory mean calibration. At logistic calibration, the average calibration slope was 1 and the average intercept was 0, showing a satisfactory weak calibration. Lastly, the examination of calibration plots showed an acceptable profile of moderate calibration (Figure 2).

## 4. Discussion

The aim of the present study was to develop and internally validate multivariable models for the prediction of FL in obese children and adolescents. Using a regression modeling strategy based on the bootstrap [27,28], we developed and internally validated two prediction models of FL, one using BMI and the other using WC as the anthropometric predictor. Both models showed good discrimination and internal calibration (Table 4 and Figure 1 and Figure 2). 

Our prediction models are obviously not intended to replace LUS. Provided that they are externally validated, these models could be used as surrogate indices of FL in obese children and adolescents in contexts where LUS is not available, as is currently suggested for adults [8]. Moreover, always after external validation, these models could be used to develop algorithms to rule in or rule in or out FL at certain values of the model score (Appendix B) [16]. The good internal calibration of these indices does not imply, of course, a similarly good external calibration [10].

Although this is the largest study performed so far in obese children and adolescents with the aim of developing multivariable models for the prediction of FL, it is not without limitations. The first limitation is that our models were developed on severely obese and adolescents and may therefore not apply to non-obese subjects. The median SDS of BMI of our cross-section of children and adolescents is in fact greater than the 99th percentile of the reference distribution [19]. Although all the predictors identified by our regression modeling strategy are in keeping with our current pathophysiological and clinical knowledge about FL [10,11,16,23,36], there is no reason to believe that the underlying prediction models will perform equally well in non-obese children and adolescents. Besides needing external validation on obese children and adolescents to assess their true performance [33,37], our prediction models will have to undergo separate evaluation in non-obese children if one plans to use them in non-obese subjects. The second limitation is that we studied only Caucasian children, the reason being that non-Caucasian individuals with obesity account for less than 2% of the patients currently followed at our Center [38]. The third limitation is that LUS, our diagnostic method, is known to offer an accurate assessment of FL only starting from an intrahepatic triglyceride content of at least 10% [39], implying a number of “false negatives”, i.e., missed cases of FL, as compared to MRI or MRS. However, LUS is presently the only feasible option to perform large studies and most of the surrogate indexes of FL currently employed in adults were developed using LUS as the reference method [40].

In addition to BMI or WC as the anthropometric predictor, the two multivariable models that we developed and internally validated had the same set of predictors: ALT, HOMA-IR, triglycerides and uric acid.

BMI and WC were entered as predictors of separate regression models to avoid multicollinearity (Table 2). Multicollinearity was not an issue when we developed FLI, which includes both BMI and WC, in the general population of Campogalliano (Modena, Italy) [16]. However, multicollinearity was present when we modelled the association between FL and potential risk factors in the general population of Bagnacavallo (Ravenna, Italy) [23]. Although this is not specified, multicollinearity between BMI and WC was likely not an issue for the development of the only prediction model of FL available to date for children, which in fact includes sex, age, BMI, and WC as predictors [12]. Quite interestingly, when BMI was replaced by WC (Table 4), the changes in the regression coefficients of the other predictors were small, reinforcing the idea that, in our cross-section of obese children, BMI and WC are interchangeable measures as the prediction of FL is concerned (Table 2). Potential advantages of BMI over WC are that: (1) its components, i.e., weight and height, are routinely measured in children of all ages; (2) its measurement is more precise than that of WC in severely obese subjects; and (3) it was recently shown to be associated with incidence and remission of NAFLD in children [41].

ALT was the only predictor chosen in all bootstrap samples in both the BMI and WC models (Table 3), confirming our previous finding of a strong multivariable association of FL with ALT [11]. ALT was highly correlated with AST and GGT (Table 2) and, to avoid multicollinearity, we choose to keep it in the models because it is more hepatospecific than AST, and, contrary to GGT, it is routinely measured as “first-level” liver enzyme. As we discussed in detail elsewhere [11], the fact that ALT is confirmed to be a component of a multivariable predictor of FL does not imply that it can be used alone to discriminate children with from those without FL.

HOMA-IR was not an independent predictor of FL in our previous study of 278 children aged 8 to 18 years with BMI > 90th percentile for age and the same was true for its components, i.e., fasting glucose and insulin, as evaluated by distinct logistic regression models [11]. However, in that study both the area under the curve of glucose and that of insulin during OGTT were independent predictors of FL [11]. Besides the larger sample size (N = 1672), the wider age (5 to 18 years) of the subjects, and the higher entry criterion for BMI (≥ 95th percentile for age) of the present study, it is possible that the more sophisticated multivariable selection of predictors used for the present analysis has allowed HOMA-IR to show its full potential as predictor of FL [28].

Interestingly, of the components of the metabolic syndrome besides WC, only triglycerides were independent predictors of FL in both the BMI and WC models, confirming our previous findings [11], those of Hosseini et al. [12], and what is more generally known about the hypertriglyceridemic waist phenotype [36].

Uric acid was identified as an independent predictor of FL in both the BMI and WC models, confirming our earlier findings [11] and the increasing evidence linking uric acid levels with NAFLD [4,14,42].

The BIF-1 of CRP was under the prespecified threshold of 66% to accept it for inclusion in both the BMI and WC models, and this was especially evident for the BMI model (Table 3). This finding is in agreement with our previous study, where CRP was not an independent predictor with FL [11].

## 5. Conclusions

In conclusion, in a large sample of obese children and adolescents, FL can be accurately diagnosed by using multivariable models based on BMI or WC, ALT, HOMA-IR, triglycerides, and uric acid. These models should undergo external validation, consisting of both discrimination and calibration [10], before being employed in clinical or research practice.

## Figures and Tables

**Table 1:** Measurements of the study subjects.

	Total	Girls	Boys
	*n* = 1672	*n* = 980	*n* = 692
Age (years)	15 (13–16)	15 (13–17)	15 (12–16)
Pubertal stage			
Tanner stage 1 (prepubertal)	194 (11.6%)	85 (8.7%)	109 (15.8%)
Tanner stage 2 (pubertal)	144 (8.6%)	48 (4.9%)	96 (13.9%)
Tanner stage 3 (pubertal)	213 (12.7%)	75 (7.7%)	138 (19.9%)
Tanner stage 4 (pubertal)	373 (22.3%)	225 (23.0%)	148 (21.4%)
Tanner stage 5 (postpubertal)	748 (44.7%)	547 (55.8%)	201 (29.0%)
Weight (kg)	96 (83–112)	93 (83–106)	103 (86–120)
Weight (SDS)	3.01 (2.47–3.57)	3.07 (2.52–3.64)	2.91 (2.37–3.43)
Height (m)	1.63 (1.56–1.69)	1.60 (1.56–1.65)	1.68 (1.58–1.75)
Height (SDS)	0.33 (−0.30–1.04)	0.29 (−0.36–1.00)	0.37 (−0.24–1.09)
BMI (kg/m^2^)	36 (32–40)	36 (32–40)	36 (32–41)
BMI (SDS)	2.92 (2.50–3.32)	2.91 (2.51–3.28)	2.92 (2.47–3.39)
Waist circumference (cm)	111 (101–122)	108 (99–118)	115 (106–126)
Large waist circumference (IDF)	1646 (98.4%)	955 (97.4%)	691 (99.9%)
ALT (U/L)	23 (16–35)	19 (15–27)	30 (21–47)
AST (U/L)	21 (17–26)	19 (16–23)	24 (20–30)
GGT (U/L)	16 (12–22)	14 (11–19)	19 (15–28)
Glucose (mg/dl)	79 (74–83)	78 (73–82)	79 (75–84)
High glucose (IDF)	10 (0.6%)	6 (0.6%)	4 (0.6%)
Insulin (μU/mL)	13 (9–18)	12 (8–18)	13 (9–19)
HOMA-IR (dimensionless)	2.4 (1.6–3.5)	2.3 (1.6–3.4)	2.6 (1.7–3.7)
Cholesterol (mg/dl)	162 (142–182)	162 (142–182)	163 (142–183)
HDL-cholesterol (mg/dL)	43 (37–51)	45 (39–53)	42 (35–48)
Low HDL (IDF)	681 (40.7%)	397 (40.5%)	284 (41.0%)
LDL-cholesterol (mg/dL)	102 (85–122)	101 (83–121)	104 (87–124)
Triglycerides (mg/dL)	87 (66–114)	83 (64–110)	90 (69–122)
High triglycerides (IDF)	163 (9.7%)	85 (8.7%)	78 (11.3%)
Uric acid (mg/dL)	6.0 (5.2–6.9)	5.7 (5.0–6.4)	6.7 (5.7–7.6)
CRP (mg/L)	0.4 (0.2–0.7)	0.4 (0.2–0.7)	0.4 (0.2–0.7)
Systolic blood pressure (mm Hg)	120 (120–130)	120 (120–130)	125 (120–130)
Diastolic blood pressure (mm Hg)	80 (70–80)	80 (70–80)	80 (70–80)
High blood pressure (IDF)	687 (41.1%)	334 (34.1%)	353 (51.0%)
Mean arterial pressure	93 (87–97)	93 (87–97)	93 (90–97)
Fatty liver	642 (38.4%)	278 (28.4%)	364 (52.6%)
Fatty liver degree			
None	1030 (61.6%)	702 (71.6%)	328 (47.4%)
Mild	250 (15.0%)	133 (13.6%)	117 (16.9%)
Moderate	300 (17.9%)	119 (12.1%)	181 (26.2%)
Severe	92 (5.5%)	26 (2.7%)	66 (9.5%)
Metabolic syndrome (IDF)	395 (23.6%)	193 (19.7%)	202 (29.2%)

Continuous variables are reported as median (50th percentile) and interquartile range (25th and 75th percentiles). Discrete variables are reported as number and proportion. Abbreviations: SDS = standard deviation scores [19]; BMI = body mass index; IDF = International Diabetes Federation [22]; ALT = alanine transaminase; AST = aspartate transaminase; GGT = gamma-glutamyltransferase; HOMA-IR = homeostasis model assessment of insulin resistance; HDL = high density-lipoprotein; LDL = low density-lipoprotein, CRP = c-reactive protein.

**Table 2:** Spearman correlation coefficients between the outcome and potential predictors.

	FL	MALE	AGE	PUB	BMI	WC	ALT	AST	GGT	GLU	INS	HOMA	HDLC	LDLC	TG	MAP	UR	CRP
fl	1.00																	
male	0.25	1.00																
age	−0.02	−0.09	1.00															
pub	−0.10	−0.30	**0.82**	1.00														
bmi	0.23	0.01	0.36	0.31	1.00													
wc	0.25	0.23	0.38	0.28	**0.77**	1.00												
alt	0.45	0.39	0.05	−0.09	0.21	0.29	1.00											
ast	0.37	0.41	−0.12	−0.24	0.03	0.12	**0.81**	1.00										
ggt	0.35	0.35	0.13	0.01	0.30	0.34	**0.61**	0.46	1.00									
glu	0.12	0.13	−0.13	−0.16	0.09	0.13	0.07	0.03	0.05	1.00								
ins	0.29	0.05	0.03	0.03	0.40	0.37	0.27	0.13	0.34	0.14	1.00							
homa	0.30	0.07	0.01	0.01	0.41	0.38	0.27	0.13	0.34	0.29	**0.98**	1.00						
hdlc	−0.16	−0.17	−0.06	−0.02	−0.22	−0.27	−0.17	−0.09	−0.20	−0.07	−0.25	−0.26	1.00					
ldlc	0.11	0.06	−0.03	−0.05	0.07	0.08	0.16	0.15	0.27	0.04	0.10	0.10	−0.05	1.00				
tg	0.21	0.10	0.07	0.02	0.19	0.21	0.25	0.17	0.31	0.00	0.34	0.32	−0.38	0.43	1.00			
map	0.11	0.14	0.30	0.22	0.41	0.42	0.19	0.06	0.23	0.06	0.25	0.25	−0.10	0.04	0.13	1.00		
ur	0.29	0.36	0.13	0.05	0.37	0.42	0.37	0.28	0.38	0.09	0.28	0.29	−0.26	0.09	0.25	0.26	1.00	
crp	0.10	−0.02	0.04	0.00	0.34	0.22	0.02	−0.04	0.16	0.04	0.14	0.14	−0.10	0.04	0.00	0.09	0.09	1.00

Abbreviations: fl = fatty liver; male = male sex; age = age; pub = pubertal status; bmi = body mass index; wc = waist circumference; alt = alanine transaminase; ast = aspartate transaminase; ggt = gamma-glutamyltransferase; glu = glucose; ins = insulin; homa = homeostasis model assessment of insulin resistance; hdlc = high-density lipoprotein cholesterol; ldlc = low-density lipoprotein cholesterol; tg = triglycerides; map = mean arterial pressure; ur = uric acid; crp = c-reactive protein. The units of measurements are the same used in Table 1. Spearman’s correlation coefficients > |0.60| are given in bold.

**Table 3:** Bootstrap inclusion fraction of the potential multivariable predictors of fatty liver (1000 bootstrap samples with replacement).

	BMI Model					WC Model			
	BIF-1	EXP-1	BIF-2	EXP-2		BIF-1	EXP-1	BIF-2	EXP-2
male	41.3	1	0.0	—	male	8.5	1	0	—
age	**95.4**	1	16.5	—	age	**91.2**	1	16.4	—
bmi	**98.1**	1	1.4	—	wc	**89.7**	1	19.0	—
alt	**100.0**	−2	89.9	−1	alt	**100.0**	−2	89.1	1
homa	**95.1**	1	51.4	2	homa	**98.6**	1	52.4	2
hdlc	63.0	1	2.9	—	hdlc	76.0	1	35.0	—
ldlc	31.1	1	20.1	—	ldlc	26.7	1	16.1	—
tg	**73.9**	1	19.1	—	tg	**69.6**	1	18.7	—
map	36.0	1	15.4	—	map	48.9	1	36.4	—
ur	**79.0**	1	15.4	—	ur	**87.4**	1	18.3	—
crp	40.0	1	21.0	—	crp	62.8	1	31.4	—

Abbreviations: BMI = body mass index; WC = waist circumference; BIF-1 = bootstrap inclusion fraction of fractional polynomial term 1; EXP-1 = exponent of fractional polynomial term 1; BIF-2 = bootstrap inclusion fraction of fractional polynomial term 2; EXP-2 = exponent of fractional polynomial term 2; male = male sex; age = age; bmi = body mass index; wc = waist circumference; alt = alanine transaminase; homa = homeostasis model assessment of insulin resistance; hdlc = high-density lipoprotein cholesterol; ldlc = low-density lipoprotein cholesterol; tg = triglycerides; map = mean arterial pressure; ur = uric acid; crp = c-reactive protein. The units of measurements are the same used in Table 1. Predictors with BIF-1 ≥ 66% are given in bold.

**Table 4:** Final multivariable models (1000 bootstrap samples with replacement).

	BMI Model	WC Model
Age (years)	−0.137 *** [−0.193 to −0.080]	−0.132 *** [−0.189 to 0.075]
BMI (kg/m^2^)	0.063 *** [0.039 to 0.086]	—
[ALT (U/l)/100]−^2^	0.036 *** [0.020 to 0.052]	0.034 *** [0.018 to 0.049]
[ALT (U/l)/100]−^1^	−0.767 *** [−0.943 to −0.591]	−0.728 *** [−0.898 to 0.557]
[HOMA-IR (dimensionless)/10]	3.583 *** [1.775 to 5.392]	3.848 *** [2.015 to 5.681]
[HOMA-IR (dimensionless)/10]^2^	−2.634 ** [−4.395 to −0.873]	−2.662 ** [−4.490 to 0.834]
Triglycerides (mg/dL)	0.004 * [0.001 to 0.007]	0.004 * [0.001 to 0.007]
Uric acid (mg/dL)	0.172 *** [0.072 to 0.272]	0.171 *** [0.070 to 0.271]
Waist circumference (cm)	—	0.022 *** [0.012 to 0.032]
Intercept	−0.533	−0.925
*n*	1672	1672
AIC	1746	1755
BIC	1794	1804
C-statistic	0.81	0.81
Cox-Snell R^2^	0.26	0.25
Nagelkerke R^2^	0.35	0.34

* *p* < 0.05, ** *p* < 0.01, *** *p* < 0.001. Values are logistic regression coefficients with bootstrapped 95% confidence intervals in brackets. Abbreviations: BMI = body mass index; ALT = alanine transaminase; HOMA-IR = homeostasis model assessment of insulin resistance; AIC = Akaike information criterion; BIC = Bayesian information criterion.

## Data Availability

The data presented in this study are available on reasonable request from the corresponding author.

## Appendix A. Transparent Reporting of a Multivariable Prediction Model for Individual Prognosis or Diagnosis (TRIPOD) Guidelines

**Table A1:** TRIPOD Checklist.

Title	1	Identify the study as developing and/or validating a multivariable prediction model, the target population, and the outcome to be predicted.	P2
Abstract	2	Provide a summary of objectives, study design, setting, participants, sample size, predictors, outcome, statistical analysis, results, and conclusions.	P2
Background and objectives	3a	Explain the medical context (including whether diagnostic or prognostic) and rationale for developing or validating the multivariable prediction model, including references to existing models.	P2
	3b	Specify the objectives, including whether the study describes the development or validation of the model or both.	P3
Source of data	4a	Describe the study design or source of data (e.g., randomized trial, cohort, or registry data), separately for the development and validation data sets, if applicable.	P3
	4b	Specify the key study dates, including start of accrual; end of accrual; and, if applicable, end of follow-up.	P3
Participants	5a	Specify key elements of the study setting (e.g., primary care, secondary care, general population) including number and location of centers.	P3
	5b	Describe eligibility criteria for participants.	P2
	5c	Give details of treatments received, if relevant (No treatment was administered)	NA
Outcome	6a	Clearly define the outcome that is predicted by the prediction model, including how and when assessed	P3
	6b	Report any actions to blind assessment of the outcome to be predicted. (The prediction models were developed retrospectively).	NA
Predictors	7a	Clearly define all predictors used in developing or validating the multivariable prediction model, including how and when they were measured.	P3
	7b	Report any actions to blind assessment of predictors for the outcome and other predictors. (The prediction models were developed retrospectively).	NA
Sample size	8	Explain how the study size was arrived at.	P3
Missing data	9	Describe how missing data were handled (e.g., complete-case analysis, single imputation, multiple imputation) with details of any imputation method. (No missing data).	P4
Statistical analysis methods	10a	Describe how predictors were handled in the analyses.	P4
	10b	Specify type of model, all model-building procedures (including any predictor selection), and method for internal validation.	P4
	10c	For validation, describe how the predictions were calculated. (Validation was internal).	P4
	10d	Specify all measures used to assess model performance and, if relevant, to compare multiple models.	P4
	10e	Describe any model updating (e.g., recalibration) arising from the validation, if done. (Internal calibration only).	P4
Risk groups	11	Provide details on how risk groups were created, if done. (Internal calibration only).	P4
Development vs. validation	12	For validation, identify any differences from the development data in setting, eligibility criteria, outcome, and predictors. (No external validation).	NA
Participants	13a	Describe the flow of participants through the study, including the number of participants with and without the outcome and, if applicable, a summary of the follow-up time. A diagram may be helpful.	P4
	13b	Describe the characteristics of the participants (basic demographics, clinical features, available predictors), including the number of participants with missing data for predictors and outcome.	P4
	13c	For validation, show a comparison with the development data of the distribution of important variables (demographics, predictors and outcome). (No external validation)	NA
Model development	14a	Specify the number of participants and outcome events in each analysis.	P4
	14b	If done, report the unadjusted association between each candidate predictor and outcome. (We directly developed a multivariable model using bootstrap selection of predictors).	NA
Model specification	15a	Present the full prediction model to allow predictions for individuals (i.e., all regression coefficients, and model intercept or baseline survival at a given time point).	P8
	15b	Explain how to use the prediction model.	App. A
Model performance	16	Report performance measures (with CIs) for the prediction model.	P8,9,10
Model-updating	17	If done, report the results from any model updating (i.e., model specification, model performance). (No updating was performed)	NA
Limitations	18	Discuss any limitations of the study (such as nonrepresentative sample, few events per predictor, missing data).	P10
Interpretation	19a	For validation, discuss the results with reference to performance in the development data, and any other validation data. (External validation not performed)	NA
	19b	Give an overall interpretation of the results, considering objectives, limitations, results from similar studies, and other relevant evidence.	P10
Implications	20	Discuss the potential clinical use of the model and implications for future research.	P10
Supplementary information	21	Provide information about the availability of supplementary resources, such as study protocol, Web calculator, and data sets.	App. B
Funding	22	Give the source of funding and the role of the funders for the present study.	P12

Abbreviations: NA = not applicable.

## Appendix B

**Table A2:** Prediction Equations.

B1. Calculation of the linear predictor for the BMI model	LP = −0.137*age + 0.063*bmi + 0.036*alt_1 − 0.767*alt_2 + 3.583*homa_1 − 2.634*homa_2 + 0.004*tg + 0.172*ur − 0.533
B2. Calculation of the linear predictor for the WC model	LP = −0.132*age + 0.022*wc + 0.034*alt_1 − 0.728*alt_2 + 3.848*homa_1 − 2.662*homa_2 + 0.004*tg + 0.171*ur − 0.925
B3. Calculation of the probability of fatty liver from the linear predictor	Probability = e^LP^/1 + e^LP^
where:	age = Age (years) bmi = Body mass index (kg/m^2^) alt_1 = [ALT (U/l)/100]−^2^ alt_2 = [ALT (U/l)/100]−^1^ homa_1 = [HOMA-IR (dimensionless)/10] homa_2 = [HOMA-IR (dimensionless)/10]^2^ tg = Triglycerides (mg/dL) ur = Uric acid (mg/dL) wc = Waist circumference (cm)
